# Mitigating effects and mechanisms of Tai Chi on mild cognitive impairment in the elderly

**DOI:** 10.3389/fnagi.2022.1028822

**Published:** 2023-01-06

**Authors:** Xin Wang, Keyi Si, Wei Gu, Xueqiang Wang

**Affiliations:** ^1^Faculty of Traditional Chinese Medicine, Naval Medical University, Shanghai, China; ^2^Department of Military Health Statistics, Naval Medical University, Shanghai, China; ^3^Department of Sport Rehabilitation, Shanghai University of Sport, Shanghai, China; ^4^Department of Rehabilitation Medicine, Shanghai Shangti Orthopaedic Hospital, Shanghai, China

**Keywords:** Tai Chi, mild cognitive impairment, elderly, cognitive function, mechanism

## Abstract

Mild cognitive impairment (MCI) is a major public health concern that endangers health and decreases the quality of life of the elderly around the world. A recent clinical guideline has recommended regular exercise (twice per week) for patients with MCI as part of an overall approach to management. Tai Chi, a form of light-to-moderate-intensity mind-body exercise, is particularly suitable for seniors. This review aims to summarize epidemiological studies related to the effects of Tai Chi on symptom remission in older adults with MCI and reveal the potential mechanisms. Evidence suggested that Tai Chi can improve cognitive functions and alleviate the accompanying symptoms of MCI in the elderly potentially by activating the expression of signals in different brain regions, altering their connectivity, increasing the brain volume, and modulating brain-derived neurotropic and inflammation factors. Studies comparing various types of Tai Chi may contribute to the identification of paradigms that have appropriate intensities and difficulty and exert good effects on older people with MCI. In addition, studies are warranted to determine the frequency and duration of training that can optimize the beneficial effects of Tai Chi on MCI.

## Introduction

Mild cognitive impairment (MCI) is a syndrome of cognitive dysfunction beyond that associated with natural aging but is not severe enough to meet the criteria for dementia. The risk of developing MCI increases significantly with age, with an annual conversion rate from 1% at age 60 to 11% at age 85 (Yesavage et al., 2002). MCI is an independent risk factor for dementia. A meta-analysis estimated that individuals with MCI were 2.3 times more likely to develop dementia than their age-matched counterparts without MCI, and the cumulative dementia incidence was 14.9% in MCI patients aged ≥65 years within a 2-year follow-up (Petersen et al., 2018).

**Figure 1:**
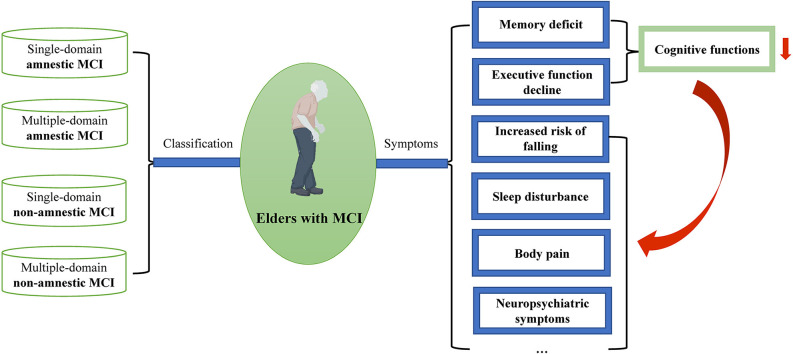
Classification and symptoms of mild cognitive impairment (MCI) in the elderly.

MCI can be divided into single/multiple-domain amnestic MCI and single/multiple-domain non-amnestic MCI (Figure 1; Petersen et al., 1997; Petersen, 2004). Amnestic MCI is predominated by a memory deficit, whereas non-amnestic MCI is characterized by disturbances of attention, information processing, psychomotor speed, language, or executive functions (Tannenbaum et al., 2012). Other symptoms may also include a deficit in gait and balance, sleep disturbance, and body pain (Kong et al., 2016; Bahureksa et al., 2017; Hu et al., 2017). Additionally, a systematic review reported that 35%–85% of patients with MCI had at least one neuropsychiatric symptom, with the most common symptoms being depression, anxiety, irritability, and apathy (Martin and Velayudhan, 2020).

To date, no pharmacological treatments specifically for MCI have been approved (Tricco et al., 2013). However, accumulating evidence from randomized clinical trials (RCTs) indicated that physical activity could benefit cognitive functions in patients with MCI (Langa and Levine, 2014; Demurtas et al., 2020). A recent clinical practice guideline has recommended regular exercise (twice per week) as part of MCI management (Petersen et al., 2018).

Tai Chi, a mind-body low-impact exercise, may offer numerous health benefits to individuals with MCI, but related results were not consistent. A systematic review and a meta-analysis of RCTs reported that Tai Chi could significantly improve memory, learning, visuospatial ability, and executive functions in patients with MCI compared with no exercise (Lim et al., 2019; Lin et al., 2021). Furthermore, Tai Chi and cognitive mixed training have shown positive effects on delaying the progression of MCI to dementia (Li et al., 2022). However, one meta-analysis found no associations between Tai Chi and global cognition and memory in older people with cognitive impairment (Wang et al., 2018). Besides, related mechanism studies are still in a nascent stage. Current evidence indicated that as a type of aerobic exercise, Tai Chi might induce some positive neurophysiological changes related to cognitive function improvements, such as increases in hippocampus size, regional cerebral blood flow, and the production of neurotrophic factors (Burdette et al., 2010; Voss et al., 2010; Suzuki et al., 2013). Moreover, some stress-related pathways (e.g., cortisol) associated with depression and anxiety alleviation might be induced by meditation, mental focus, and deep breathing in Tai Chi (Wayne et al., 2014).

Given the light to moderate intensity [about three metabolic equivalents of task (METs)], Tai Chi has attracted people worldwide in pursuit of health and longevity, especially the frail elderly who experience a functional decline (Hui et al., 2016). This article aimed to give a comprehensive review about the impact of Tai Chi on MCI in the elderly and the potential mechanisms.

## Amelioration of The Clinical Symptoms of MCI Through Tai Chi in The Elderly

### Improve memory function

Amnestic MCI is a subtype of MCI manifesting as a decline in various types of memory (Leyhe et al., 2009; Costa et al., 2011; Tuena et al., 2021). The prevalence of amnestic MCI in people aged ≥60 was 7.4% in South Italy and 17.1% in China (Li et al., 2020; Caffo et al., 2022). Older patients with amnestic MCI are more likely to progress to Alzheimer’s dementia than those with non-amnestic MCI (Jungwirth et al., 2012). Tai Chi, which calls for a substantial cognitive effort to learn new skills by remembering and performing movements, might prevent or alleviate memory loss in older adults with MCI (Lin et al., 2021).

An RCT from Thailand reported that the increase in logical memory delayed recall score was more pronounced in the 10-form Tai Chi group (three times per week for 6 months) than in the educational control group [mean difference: 0.43; 95% confidence interval (CI): 0.2, 0.7; Sungkarat et al., 2018]. An RCT from China also found that the Mattis memory score was enhanced in the Tai Chi group (three times per week for 40 weeks) in contrast to that in the other three groups (walking, social interaction, or no intervention; Mortimer et al., 2012). In another RCT from China, 389 older participants with MCI were randomized into either the 24-form simplified Tai Chi group or the exercise group (stretching and toning), and both their delayed recall and subjective memory complaints significantly improved after 5 months of intervention, but the positive effect only remained for delayed recall at 1-year post-intervention (Lam et al., 2011, 2012). In contrast, there are also some studies finding no associations between Tai Chi and memory improvement in older people with cognitive impairment compared with health education (Wang et al., 2018). The authors speculated that the beneficial effects of Tai Chi might not be apparent until enough training (60 min per time and three times per week for at least 12 weeks) had been done. In addition, a study found that Taoist Tai Chi + memory intervention program (MIP) was not superior to MIP alone in memory performance (Fogarty et al., 2016).

### Improve executive function

The executive function refers to a set of cognitive abilities that help an individual plan, monitor, and achieve a goal, mainly including inhibition and interference control, working memory, and cognitive flexibility (Diamond, 2020). Elderly people with MCI are likely to have some defects in executive functions, which might be alleviated by exercises, such as Tai Chi (Law et al., 2020). A systematic review and meta-analysis found that Tai Chi was effective in improving the executive functions of processing speed, attention, and working memory compared with either non-active (health education or no intervention) or active controls (walking or endurance, resistance/strength, and flexibility exercises; Wayne et al., 2014). However, the number of studies was very limited (Tai Chi vs. non-active controls: *N* = 4; Tai Chi vs. active controls: *N* = 2). Apart from studies in the meta-analysis, an individual trial showed that 6 months of 10-form Tai Chi training (three times per week) was effective in improving the mental switching component of executive function in older adults with amnestic MCI (Sungkarat et al., 2018). The beneficial effects on executive function in exergaming-based Tai Chi group (modified Yang style), traditional Tai Chi group (simplified 24-form Yang style), and untrained controls were compared (Liu et al., 2022). During the Tai Chi exergames, participants imitated a virtually-presented Tai Chi instructor and made real-time adjustments in response to immediate feedback. These two Tai Chi groups (three times per week for 12 weeks) had similar improvements in executive functions, significantly stronger than the controls. Two RCTs assessing the effects of 24-form or 10-form Tai Chi on components of executive function showed significant improvement in Trail Making Test (task switching) but not the digit span forward and backward test (information updating and monitoring), indicating that the intervention effect of Tai Chi might be specific to certain executive components (Lam et al., 2011; Sungkarat et al., 2018).

### Improve global cognitive function

Tai Chi is a light-to-moderate-intensity aerobic exercise, which may offer benefits to global cognitive functions (Bao and Liu, 2019). A 48 times 8-form Tai Chi training within 3 months can significantly improve the MOCA-P (the Peking Union Medical College version of Montreal cognitive assessment) total score of the elders with MCI, especially in its sub-project score (visual space function and long delayed recall) compared with the control group (a 30-min health education about MCI prevention and a related handbook; Wang and Sheng, 2016). The higher the MOCA-P score, the better the global cognitive function is. Systematic reviews demonstrated that compared with non-intervention controls or other active interventions (adapted physical activity and health education, stretching and relaxation exercise, walking, etc.), 12 weeks to 1 year of Tai Chi training could yield small to moderate clinically relevant improvements in global cognitive functions for cognitively impaired older adults (Wayne et al., 2014; Lim et al., 2019; Zhou et al., 2022). A pilot RCT compared the effects of 24-form Yang-style Tai Chi vs. conventional exercise with similar intensity on global cognitive function in adults aged ≥50 years with MCI (Yu et al., 2022). The results indicated that Tai Chi resulted in earlier (12 weeks vs. 24 weeks) and more significant improvements in cognitive flexibility. Notably, Tai Chi had additional positive effects on global cognitive function in comparison to cognitive training alone after 12 months of intervention, but the beneficial effects in both groups gradually disappeared after they stopped training, and only those who continued to receive the combined training maintained their cognitive performance after another 12 months (Li et al., 2022). Therefore, prolonged training is required to delay cognitive decline. A meta-analysis revealed that as the duration of Tai Chi increased from 20–40 min to 60 min, profound improvement in global cognitive function would be obtained. However, one study found that the cognitive function was not differentially improved in the Taoist Tai Chi + MIP group relative to the MIP-only group over the shorter term (10 weeks) and longer-term (22 weeks; Fogarty et al., 2016). This might be attributed to the instability of the test or the relatively large difference in baseline cognitive functions between the groups.

### Reduce the risk of falls

Good performance in maintaining balance and gait not only relies on the health of muscles and bones but also on that of some cognitive functions, such as executive-attentional functions, visuospatial abilities, and memory (Amboni et al., 2013). Degenerated muscles and bones and cognitive impairment often coexist in older adults, increasing the risk of falls (Tamura et al., 2018). A study reported that older people with cognitive impairment were four times more likely to fall than those without these issues (Tinetti et al., 1988).

Tai Chi might be an effective strategy for preventing falls in the elderly with MCI possibly by improving their somatic and cognitive functions related to balance, muscle strength, physical endurance, postural control, and flexibility (Wehner et al., 2021). A meta-analysis summarized that Tai Chi might reduce the incidence of falls by 43% over a short time (<12 months) in older and at-risk adults (Lomas-Vega et al., 2017). Sungkarat et al. (2017) assessed the effect of a combination of center- and home-based 10-form Tai Chi training on the risk of falls in older adults with MCI using various indicators, including knee proprioception, knee extension strength, hand reaction time, postural sway, and Physiological Profile Assessment fall risk index. These indices significantly improved after a 15-week intervention (three times per week). In addition to field training, the effects of Tai Chi online courses on the risk of falls have been explored during the COVID-19 pandemic. Liu et al. (2022) found that the exergaming-based Yang-style Tai Chi and traditional Yang-style Tai Chi groups had comparable improvements in gait performance and cognitive-dual-task cost, both of which were better than those of the untrained control group. Inconsistently, Li et al. (2021) found that though the home-based Tai Chi group performed better on tests of balance, chair stands, and gait performance than the stretching group, no significant reduction in falls (incidence rate ratio: 0.58; 95% CI: 0.32, 1.03) was observed possibly because of the small sample size (*N* = 15 for each group). Besides, a low-quality study reported that the Taoist Tai Chi + MIP on gait or balance showed no preferential benefit to the elders with MCI than the pure memory training (Fogarty et al., 2016).

### Improve sleep quality

Compared with a normal population, MCI patients are more likely to experience sleep disturbances (18.3%–45.4% vs. 10.9%–23.3%), manifesting as greater wake after sleep onset, lower sleep efficiency, and longer rapid eye movement sleep latency (Da, 2015; D’Rozario et al., 2020). Some studies have suggested a bidirectional relationship between sleep problems and cognitive decline (Guarnieri and Sorbi, 2015). Specifically, circadian dysregulation might precede MCI and accelerate its progress to Alzheimer’s disease, and different patterns of sleep disorders might result from specific dementias, depending on the areas affected in the brain (Tranah et al., 2011; Roth, 2012).

Tai Chi has been proposed as an effective intervention to mitigate sleep problems in older adults, but the evidence is limited in patients with MCI. In a pilot RCT, older adults with cognitive impairment who attended the 10-form Tai Chi sessions twice a week for 2 months had prolonged sleep duration (+48 min) and increased sleep efficiency (+9.1%) after 6 months, significantly better than those of the untrained group (Chan et al., 2016). A systematic review found that generally healthy older adults had the greatest number of significant improvements in various sleep outcomes after they performed moderate intensity exercise (e.g., Baduanjin, Tai Chi, and yoga) three times per week for at least 3 months (Vanderlinden et al., 2020). Regular exercise, especially Tai Chi training involving deep breathing and meditation, may promote relaxation and raise body temperature in ways that are helpful for initiating and maintaining sleep (Montgomery and Dennis, 2002).

### Reduce body pain

MCI in older people is often accompanied by inflammation and chronic pain. A prospective cohort study demonstrated that the prevalence and severity of chronic pain were independently influenced by cognitive measures (attention, visual memory, and executive function) and affective factors (state anxiety, passive coping strategies, and depression; Attal et al., 2014). Systematic reviews and meta-analyses of RCTs demonstrated that Tai Chi was an effective intervention for relieving low back pain and pain caused by osteoporosis, osteoarthritis, and fibromyalgia (Kong et al., 2016). Lauche et al. (2016) showed the effectiveness of 12 weeks of 13-form Yang-style Tai Chi training in improving chronic nonspecific neck pain relative to non-trained controls. A pilot study found that compared with light physical exercise program, a 12-week 8-form Yang-style Tai Chi training program could significantly lower pain severity and pain interference in older adults with chronic multisite pain (You et al., 2018). The valid duration of Tai Chi for osteoarthritis pain relief was at least 6 weeks as suggested by a systematic review and meta-analysis (Kong et al., 2016). In 2017, a clinical practice guideline from the American College of Physicians recommended patients with chronic low back pain to select Tai Chi or other mind-body exercise as a nonpharmacologic treatment (Qaseem et al., 2017). However, related evidence is limited and has low or moderate quality. Overlaps have been found among the brain regions responsible for pain modulation and cognition, such as the amygdala, prefrontal cortex, and hippocampus (Attal et al., 2014; Shen et al., 2021). When Tai Chi improves cognitive functions through altering these regions, some beneficial effects on pain relief may occur.

### Relieve internal bad sentiment and improve the quality of life

Even though senior people with MCI can perform daily activities, they may still face some cognitive, social, or psychological problems that can make them feel anxious and upset, and thus their quality of life is reduced. Older adults with MCI who have low income, depressive symptoms, or poor sleep quality are likely to report low quality of life (Song et al., 2019). Eight weeks of the 10-form Tai Chi training has been linked to improvement in the mental health component of quality of life in older adults with cognitive impairment (Chan et al., 2016). Researchers speculated that such benefits might be attributed to more social interaction and companionship offered by Tai Chi. After a 16-week Yang-style simple Tai Chi training, older patients with MCI scored higher in some subscales of Short-form 12, a measure of the quality of life, than untrained controls (Siu and Lee, 2021). Reduction in depression and anxiety, alleviation of body pain, exaltation on activities of daily living, and improvement in sleep quality may somehow mediate the effects of Tai Chi on the quality of life in patients suffering from MCI (Siu and Lee, 2018; Farhang et al., 2019).

**Figure 2:**
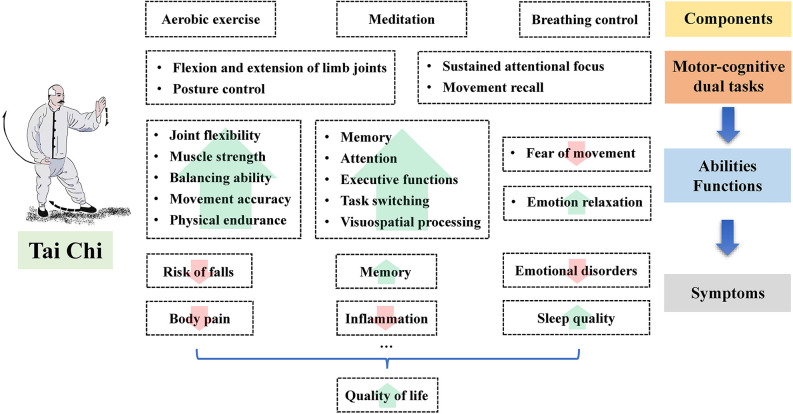
The potential benefits of Tai Chi on the symptoms of mild cognitive impairment in the elderly.

As previously mentioned, many epidemiological studies have identified the beneficial effects of Tai Chi on symptoms of MCI in the elderly, including memory deficit, executive function decline, sleep disturbance, body pain, and emotional disorders (Table 1). The soft movement and the postural control in Tai Chi may improve the flexibility of the limb joints and the stability of the posture, while the combination of aerobic exercise, meditation, and breathing control may alleviate emotional distress and improve some cognitive functions, such as the memory function and the executive function (Figure 2).

**Table 1:** Characteristics of studies related to the effects of Tai Chi on mild cognitive impairment in the elderly.

**Studies**	**Country**	**Diagnostic criteria**	**Participants (IG/CG)**	**Age, mean (SD), year**	**Intervention**	**Frequency and duration of Tai Chi**	**Follow-up**	**Adverse event**	**Outcomes/Measurements**
Bao and Liu (2019)	China	Amnestic MCI, Global Deterioration Scale: 2–3; CDR score: 2; ADL ≤26	31/31	IG: 68.22 (9.84); CG: 65.62 (9.34)	IG: 24-form Tai Chi; CG: health education	Twice a day, 3 days a week for 6 months	6 months	Not reported	Tai Chi group had higher MMSE and MOCA scores, shorter P300’s incubation period, and higher volatility than the control group (*P* < 0.05).
Birimoglu and Deveci (2021)	Turkey	MCI (MMSE and MoCA <25)	20/22	IG: 74.21 (6.93); CG: 74.21 (6.93)	IG: Yang style Tai Chi; CG: not subjected to any physical practice	35–40 min/session, twice a week for 3 months	3 months	Not reported	The scores of cognitive adaptations and changes in level subscales of the fall behavioral scale were significantly increased in the Tai Chi group (*P* < 0.01).
Chan et al. (2016)	China	MCI (MMSE 13–26)	27/25	IG: 78.4 (7.1); CG: 82.2 (6.7)	IG: 10-form Tai Chi; CG: usual activities	60-min session, twice a week for 2 months	6 months	None	Tai Chi group had significantly better results in the Chinese Pittsburgh Sleep Quality Index global score (*P* = 0.004), sleep duration (*P* = 0.003), habitual sleep efficiency (*P* = 0.002), and the Short-form 12 mental health component (*P* < 0.001).
Fogarty et al. (2016)	Canada	Amnestic MCI, MMSE	22/18	CG: 71.55 (9.33); CG: 72.61 (5.78)	IG: Taoist Tai Chi + MIP; CG: MIP	2 × 90 min for 10 weeks	3 months	Not reported	Both groups significantly increased their memory strategy knowledge and use, ratings of physical health, processing speed, everyday memory, and visual attention. No preferential benefit was found for individuals in the MIP + Tai Chi group on cognition, gait, or balance measures.
Kasai et al. (2010)	Brazil	MCI (RBMT ≤10)	13/13	IG: 73.54; CG: 74.54	IG: Yang style Tai Chi; CG: not practice Tai Chi	60-min per session, twice per week for 6 months	6 months	Not reported	Tai Chi group had significant improvement in the RBMT and the SMC (*P* = 0.007 and *P* = 0.023).
Lam et al. (2011)	China	CDR 0.5 or amnestic MCI	135/194	IG: 77.2 (6.3); CG: 78.3 (6.6)	IG: 24-form simplified Tai Chi; CG: stretching and toning exercise	3 × 30 min/week for 8–12 weeks + video CD of Tai Chi + monthly refresher lessons for 5 months	5 months	One fall with minor injury in CG	Tai Chi group had significantly better performance in visual spans and CDR sum of boxes scores (*P* < 0.001) and lower risk of dementia (*P* < 0.05).
Lam et al. (2012)	China	CDR 0.5 or amnestic MCI	92/169	IG: 77.2 (6.3); CG: 78.3 (6.6)	IG: 24-form simplified Tai Chi; CG: stretching and toning exercise	3 × 30 min/week for 4–6 weeks + video CD of Tai Chi + monthly refresher lessons for 12 months	1 year	Death: 1 in IG and 2 in CG; fall with bone fracture: 1 in IG and 1 in CG	Tai Chi group had a lower risk of developing dementia (*P* = 0.04) and a better preservation of CDR sum of boxes scores (*P* = 0.04). Tai Chi group had greater improvement in delay recall (*P* = 0.05) and Cornell Scale for Depression in Dementia scores (*P* = 0.02) in completers-only analyses.
	China	MCI (20 ≤MMSE ≤30)	22/24	IG: 75 (11); CG: 77 (10)	IG: Tai Ji Quan; CG: usual daily physical activities	60-min per session, twice per week for 14 weeks.	14 weeks	None	Tai Chi group had significant improvement on MMSE (*P* < 0.001), physical performance, and balance efficacy measures (*P* < 0.05).
Li et al. (2021)	China	MCI (CDR ≤0.5 and MMSE ≥24)	15/15	IG: 76.13 (6.2); CG: 76.2 (6.3)	IG: Tai Ji Quan; CG: stretching	60-min virtual exercise sessions *via* Zoom, twice weekly for 6 months	6 months	No serious intervention-related adverse events	Tai Chi group did not reduce falls or the number of fallers, but performed consistently better in balance (30-s chair stands and Timed Up and Go under single-task and dual-task conditions).
Li et al. (2022)	China	MCI (detailed medical history, neurological examinations, (CDR = 0.5) and MTA)	48/51	IG: 65.6 (5.6); CG: 65.5 (7.2)	IG: Tai Chi and cognitive training; CG: cognitive training	2 × 120 min/week for 12 months; a subgroup of IG received training for another 12 months	24 months	Not reported	Tai Chi enhanced global cognitive function in MCI. Tai Chi and cognitive mixed exercise showed positive effects on delaying cognitive decline.
Liu et al. (2022)	China	MCI based on Petersen’s criteria	16 + 17/17	IG: 74.6 (6.1) and 73.2 (6.3); CG: 73.4 (6.5)	IG: exergaming-based/traditional Tai Chi (24-form Yang style); CG: usual daily physical activities	3 × 50 min/week for 3 months	3 months	None	The exergaming-based and traditional Tai Chi groups performed better than the control group on the Chinese version of the Stroop Color and Word Test, the TMT (A and B), the one-back test, gait speed, and dual-task cost of gait speed in cognitive dual-task conditions. Only the exergaming-based Tai Chi group experienced beneficial effects for the Montreal Cognitive Assessment.
Siu and Lee (2021)	China	MCI (MMSE 19–28)	80/80	IG: 60–74 (78.8%), 75 or above (21.3%); CG:60–74 (55%), 75 or above (45%)	IG: 24-form Yang-style Tai Chi; CG: usual activities	60 min per session, 2 sessions per week for 16 weeks	17–18 weeks	Not reported	Tai Chi group performed better in the physical health component (*P* = 0.036), the mental health component (*P* = 0.014), as well as several subscales of SF-12, namely, the role-physical (*P* = 0.044), the bodily pain (*P* < 0.001) and the vitality (*P* = 0.004) subscales.
Sungkarat et al. (2017)	Thailand	Amnestic MCI, MMSE ≥24 and MoCA <26	33/33	IG: 68.3 (6.7); CG: 67.5 (7.3)	IG: 10-form Tai Chi; CG: health education	3-week center-based + 12-week home-based Tai Chi (50 min per session, 3 times per week)	15 weeks	One ill health, one study-unrelated ankle fracture	Tai Chi group had significantly better performance on Logical Memory, Block Design (visuospatial ability), TMT (B-A; executive function), composite PPA score (fall risk), and PPA parameter scores (knee extension strength, reaction time, postural sway, and lower limb proprioception).
Sungkarat et al. (2018)	Thailand	Amnestic MCI, MMSE ≥24 and MoCA <26	33/33	IG: 68.3 (6.7); CG: 67.5 (7.3)	IG: 10-form Tai Chi; CG: health education	3-week center-based + 6 months home-based Tai Chi (50 min per session, 3 times per week)	27 weeks	One ill health, one study-unrelated ankle fracture	Tai Chi group had significantly better performance on Logical Memory and TMT (B-A), and higher plasma BDNF levels. No significant difference on Block Design scores, Digit Span forward/backward scores (executive function), and plasma TNF-α and interleukin-10 levels were observed.
Tsai et al. (2015)	USA	Moderate, mild, or subtle MCI and knee osteoarthritis (18 ≤ MMSE ≤28 and VDS ≥2 or WOMAC ≥3)	28/27	79	IG: 12-form Sun-style Tai Chi; CG: instructor-led educational activities	3 sessions per week for 20 weeks	20 weeks	None	The VDS and observed pain behaviors were significantly better in the Tai Chi group (*P* = 0.008–0.048).
Wang and Sheng (2016)	China	MCI (MoCA-P ≤25)	43/49	IG: 6–70: 2, 70–80: 30, 80–85: 11; CG: 65–70: 2, 70–80: 30, 80–85: 17	IG: 8-form Tai Chi; CG: health education	At least 40 min each time, at least 4 times a week for 3 months	3 months	One case of accidental fall	The total scores of MoCA-P and the scores of visual space function and long-delayed recall were higher in the Tai Chi group (*P* < 0.01).
Xu et al. (2020)	China	MCI (MoCA-Hong Kong: 19–21)	6/6	IG: 76.43 (4.47); CG: 70.67 (4.23)	IG: 24-form simplified Tai Chi; CG: health advice	12-week training + 12-week practice (30 min per session, 3 times per week)	6 months	One leg pain	Tai Chi group had a significantly lower cost of health service utilization and better performance in DAD score and GAS-20 (*P* < 0.05).
Young (2020)	China	Mild-stage dementia (MMSE ≥18)	41/39	IG: 80.05 (6.17); CG: 80.25 (6.33)	IG: 8-form Tai Chi; CG: usual activities	60 min per session, twice a week, with a total of 14 sessions	7 weeks	Not reported	Tai Chi group was more effective in improving the Dementia Rating Scale and MMSE scores (*P* < 0.01).
Yu et al. (2022)	China	MCI (below the 7th percentile of the normative data using the MoCA-Hong Kong)	10/12	IG: 67.3 (4.2); CG: 67.2 (6.8)	IG: 24-form Yang-style Tai Chi; CG: conventional exercise	3 sessions of 60-min training per week for 6 months	6 months	None	Tai Chi group showed greater improvements in MoCA-Hong Kong score than the exercise group at 12 weeks (*P* < 0.001), but the difference was no longer significant at 24 weeks (*P* = 0.061).

Note: ADL, Activities of Daily Living; BDNF, brain-derived neurotrophic factor; CDR, Clinical Dementia Rating Scale; CG, control group; CPQSI, the Chinese Pittsburgh Sleep Quality Index; DAD, the Disability Assessment for Dementia; GAS-20, the Geriatric Anxiety Scale with a maximum score of 20; GDS, Geriatric Depression Scale; HK-MoCA, Montreal Cognitive Assessment Hong Kong version; IG, intervention group; MCI, mild cognitive impairment; MIP, memory intervention program; MMSE, Mini-Mental State Examination; MoCA, Montreal Cognitive Assessment; MoCA-P, the Peking Union Medical College Version of Montreal Cognitive Assessment; MTA, The Visual Rating Medial Temporal Atrophy Score; PPA, Physiological Profile Assessment; RBMT, Rivermead Behavioral Memory test; SD, standard deviation; SMC, subjective memory complaints; TC, Tai Chi; TNF, tumor necrosis factor; TMT, Trail-Making Test; VDS, Verbal Descriptor Scale; WOMAC, the Western Ontario and MacMaster pain scale.

## Mechanisms Underlying The Effects of Tai Chi on Cognitive Functions

### Alter the functional connectivity of different brain regions

The human brain is a large network composed of structurally and functionally interconnected components (Fam et al., 2020). It can be artificially divided into different regions (lobes) in charge of various functions. Accumulating neuroimaging studies have demonstrated that MCI is a disorder characterized by brain dysconnectivity. The beneficial effects of Tai Chi on cognitive functions in older patients with MCI might be mediated by altering the connectivity of the brain network.

The prefrontal cortex, a region controlling the highest levels of cognitive and emotional processes, is extremely vulnerable to aging, and declines in these processes manifest as disrupted connectivity among brain regions (Jones et al., 2019; Yan and Rein, 2022). Research discovered that the resting-state functional connectivity between the medial prefrontal cortex and bilateral hippocampus increased significantly after 12 weeks (5 days per week) of Tai Chi training; the increase was associated with corresponding improvement in memory functions measured by the Wechsler Memory Scale (Li et al., 2014). Similarly, another study reported that a multimodal intervention comprising cognitive training (181-h sessions), Tai Chi (three times per week for 6 weeks), and group counseling (once per week) was efficacious in improving the resting-state functional connectivity between the medial prefrontal cortex and the medial temporal lobe and the strength of the connectivity was positively correlated with changes in cognitive performance assessed by the Category Fluency Test (Tao et al., 2016; Tao et al., 2017a). Hui et al. (2016) monitored the functional connections of different brain regions among experienced Tai Chi practitioners and untrained controls during resting and movement states. They found that Tai Chi could induce the activation of the prefrontal cortex, motor cortex, and occipital cortex, improve the connections among them in myogenic activity, sympathetic nervous system, and endothelial cell metabolic activities, and increase cerebral blood flow, which was associated with improvement in cognitive functions. Other studies have shown that Tai Chi could increase connectivity between the left middle frontal gyrus and the left superior parietal lobule, between the posterior cingulate cortex and the right putamen/caudate, or between the left prefrontal cortex and the right sensorimotor area and reduced connectivity among the bilateral dorsolateral prefrontal cortex, the left superior frontal gyrus, and the anterior cingulate cortex (Tao et al., 2017b; Liu J. et al., 2019; Wang and Lu, 2022).

The status of brain region connections is closely related to the efficiency of information transfer (Sun et al., 2014). Compared with general aerobic exercise, 8 weeks of Tai Chi training could significantly enhance the nodal clustering coefficient of the left thalamus, an indicator of the ability of information processing and transmission between this region and adjacent brain regions (Cui et al., 2021). This was also reflected as higher cognitive flexibility (smaller reaction time difference between the heterogeneous and homogeneous tasks) in Tai Chi practitioners. Given that the thalamus plays an important role in attention, working memory, and sleep, it is reasonable why Tai Chi can help attenuate the progressive decline of cognitive functions (Mitchell et al., 2014).

Elderly people with MCI are more likely to develop neuropsychiatric disorders, such as anxiety and depression. Tai Chi might be efficacious in alleviating negative feelings by strengthening the fronto-striatal functional connectivity or weakening the connectivity between the dorsolateral prefrontal cortex and the middle frontal gyrus (Liu et al., 2018, 2020).

### Increase the activation of the prefrontal cortex

The prefrontal cortex, a brain region involved in many cognitive functions, including working memory, sensory attention, abstraction, planning, value-based decision-making, and motor control, is vulnerable to neurodegeneration associated with aging (Chafee and Heilbronner, 2022). Reduced activation in the dorsolateral prefrontal cortex during memory retrieval may lead to adverse memory performance (Uemura et al., 2016). Tai Chi might reduce the degeneration of the prefrontal cortex caused by aging by increasing its activation. Oxyhemoglobin, a reliable indicator of changes in regional cerebral blood flow, can be used to reflect cortical activation during movement (Perrey, 2008). Lu et al. (2016) reported a case in which the prefrontal oxyhemoglobin level was monitored when a Tai Chi master was asked to perform Tai Chi and cycling on two separate days. They found a greater increase in oxyhemoglobin and total hemoglobin levels (sum of oxygenated and deoxygenated hemoglobin) during the Tai Chi routine, suggesting that the subject had greater prefrontal neuronal activation during Tai Chi exercise than that during cycling, though these two activities had similar intensities (2.6 METs for Tai Chi and 2.3 METs for cycling). This research speculated that the additional activation was due to the mental involvement unique to Tai Chi. Some studies found increased prefrontal activation assessed by functional magnetic resonance imaging or functional near-infrared spectroscopy and increased resting-state activity in the dorsolateral prefrontal cortex evaluated by the fractional amplitude of low-frequency fluctuations in the Tai Chi group (Wu et al., 2018; Xie et al., 2019; Wang S. et al., 2021).

### Increase gray matter density and regional homogeneity in the hippocampus

The hippocampus, a region that plays a vital role in learning, memory formation and consolidation, and emotion regulation, is located within the medial temporal lobe of the brain (Bartsch and Wulff, 2015). A series of alterations in the aging hippocampus, including decreased volume and neuronal density, increased neuroinflammation, and altered intracellular signaling, has been associated with cognitive decline (Driscoll et al., 2003; Bettio et al., 2017). Compared with controls, Tai Chi practitioners were reported to have increased gray matter densities, higher regional homogeneity in the hippocampus, and enhanced memory-related test scores; this finding implied that Tai Chi improves memory functions by altering the hippocampus at structural and functional levels (Yue et al., 2020a). Specifically, participants who regularly performed Tai Chi for at least 6 years had higher gray matter densities in the left hippocampus and left parahippocampal gyrus than those who walked for exercise. Regional homogeneity is a data analysis method of resting-state functional magnetic resonance imaging technique and mainly reflects the consistency of the spontaneous activities of local neurons (Liu et al., 2021). This study also found significantly higher regional homogeneity activation in the hippocampus and a positive relationship between this factor and memory performance in the Tai Chi group, which is consistent with the results of another study with 6 weeks of Tai Chi intervention (Zheng et al., 2015).

### Improve the efficiency and microstructure of brain white matter

The white matter serves a critical role in cognitive functions because it connects gray matter regions throughout the brain (Filley and Fields, 2016). The reduced integrity of the white matter during aging is related to a decline in memory, executive function, and general cognition (Coelho et al., 2021). Older patients with MCI and memory deficit exhibited decreased white matter integrity for the bilateral dorsal frontal-striatal tract, left anterior thalamocortical radiations-ventral part, and corpus callosum connecting the bilateral inferior parietal lobule and ventral prefrontal regions (Chang et al., 2021). Compared with older adults who regularly performed walking as exercise for at least 6 years, older adults who performed Tai Chi for a similar period had a smaller small-world attribute (sigma), which indicated higher efficiency of information transfer in the white matter network (Yue et al., 2020b). This attribute is associated with enhanced performance of the updating function in working memory. Apart from the statistical evidence of a small-world network, a study used diffusion tensor imaging to explore the microstructure of the brain white matter of Tai Chi practitioners and their sedentary counterparts (Yao et al., 2019). The results showed that the fractional anisotropy in the splenium of the corpus callosum was significantly higher in the Tai Chi group than in the control group, suggesting the improved integrity of the white matter. Additionally, a meta-analysis of RCTs investigating the role of Tai Chi on cardiorespiratory fitness in elderly people indicated that Tai Chi can significantly increase the maximum rate of oxygen consumption (VO_2_max). Meanwhile, white matter microstructure mediates the association of VO_2_max with spatial working memory performance (Tan et al., 2022). In summary, Tai Chi might improve cognitive functions by slowing down white matter degeneration.

### Increase the brain volume and cortex thickness

Older adults with MCI often exhibit increased brain atrophy rate. Patients with MCI accompanied by hippocampal atrophy, medial temporal lobe atrophy, or entorhinal atrophy are at a high risk of developing Alzheimer’s disease (Li et al., 2016). Decreases in the volumes of different brain regions might lead to impairment in cognitive functions. For instance, a decrease in the hippocampal volume has been linked to poor episodic memory, working memory, and executive functioning (Hardcastle et al., 2020). Mortimer et al. (2012) reported that after 40 weeks of Tai Chi training, the whole brain volume increased by 0.47%, significantly higher than that in the walking group (−0.15%) or no intervention group (−0.24%); this finding might partially explain cognitive improvements related to verbal learning, attention, and memory in the Tai Chi group. Compared with general aerobic exercise, 8 weeks of Tai Chi tends to have a stronger effect on brain plasticity, as evidenced by an increase in gray matter volume in the left middle occipital gyrus, left superior temporal gyrus, and right middle temporal gyrus (Cui et al., 2019). These regions are highly correlated with visual information processing, emotion regulation, language/semantic processing, and memory encoding and retrieval (Pico-Perez et al., 2017; Teng et al., 2018; Liu et al., 2021). In addition, increased gray matter volume has been found in the insula, medial temporal lobe, putamen, thalamus, and hippocampus in Tai Chi practitioners (Tao et al., 2017c; Liu S. et al., 2019).

Apart from an increase in brain volume, an increase in cortical thickness was observed in some specific brain regions in the Tai Chi group, such as the precentral gyrus, the middle frontal sulcus, the superior temporal gyrus, and the medial occipito-temporal sulcus, which are responsible for motor execution, emotion and cognition integration, and special navigation (Wei et al., 2013). Similar effects can be induced by meditation or aerobic exercise; hence, these activities might have common mechanisms for brain reshaping (Lazar et al., 2005; Rogge et al., 2018).

### Change brain event-related potentials

An ERP refers to an electrical brain response time-locked to a particular stimulus or event, which can be recorded *via* electroencephalography (Aaronson, 2021). ERPs are commonly used in assessing cognitive processing at a high temporal resolution (Helfrich and Knight, 2019). In this context, ERPs as indicators are more sensitive than behavioral reactions (Fong et al., 2014). P3b and P3 amplitudes are indices of neural resource and cognitive processing capability allocation and have large amplitudes reflecting enhanced processing capability; P3 latency reflects stimulus evaluation time or processing speed, with shorter latency indicating superior performance (Gao et al., 2013). Under a natural condition, P3 amplitude decreases regularly with increasing age at a rate of 0.18 μV per year, whereas its latency increases at a rate of 1.36 ms per year (Picton et al., 1984). Tai Chi might mitigate such a trend. A cross-sectional study reported that Tai Chi practitioners had larger P3b amplitudes than sedentary controls; this result was consistent with that of another study, which showed a larger P3 amplitude in older adults practicing Tai Chi in a task-switching task (Fong et al., 2014; Hawkes et al., 2014). This phenomenon implies that Tai Chi practitioners allocate more attention or other resources to the task given by researchers than individuals that do not perform Tai Chi and thereby have better cognitive performance. The second study found shorter P3 latency in the Tai Chi group than in sedentary counterparts, indicating the faster processing speed of the practitioners. These findings are consistent with the “compensation hypothesis,” which suggests that age-related cognitive deterioration can be compensated by the frontal activation of neural networks through learning new skills or exercise (Fong et al., 2014).

### Increase concentrations of brain-derived neurotrophic factor

Brain-derived neurotrophic factor (BDNF) is a protein that can regulate a wide range of activities essential for the development and maintenance of normal brain functions (Colucci-D’Amato et al., 2020). It is considered a mediator in the association between an external stimulus (e.g., exercise and enriched environment) and improved neurogenesis and synaptic plasticity in the hippocampus and prefrontal cortex, which is critical for learning and memory (Numakawa et al., 2018). Results of a trial showed that the aerobic exercise-induced upregulation of serum BDNF was associated with increased hippocampal volume and improved memory function (Erickson et al., 2011). An RCT demonstrated that 6 months of the 10-form Tai Chi practice was effective in upregulating plasma BDNF along with improving memory and mental switching in older patients with amnestic MCI (Sungkarat et al., 2018). A similar phenomenon was also observed in another study with 10 weeks of Tai Chi intervention (Solianik et al., 2021).

### Decrease plasma amyloid beta (Aβ) and total tau (t-tau) protein levels

The hallmark neuropathological substrates for MCI are β-amyloid (Aβ) plaques and intracellular tau neurofibrillary tangles (NFTs; Chandra et al., 2019). Aβ peptides have been demonstrated to lead to lipid peroxidation in amnestic MCI brains (Butterfield and Boyd-Kimball, 2018). The severity of cognitive symptoms and disease duration are positively correlated with the existence and the amount of hyperphosphorylated tau-based NFT pathology (Gómez-Isla et al., 1997). A mice study found that 8 weeks of aerobic exercise (treadmill) could alleviate memory impairments by reducing tau hyperphosphorylation in the cerebral cortex (Jeong and Kang, 2018). Tai Chi is a type of aerobic exercise and may have a similar effect. Reduced Aβ1–42 was considered evidence of amyloid deposition in the brain (Harris et al., 2015). A positive association was observed between the plasma level of Aβ1–42 and global cognition and hippocampal volume (Poljak et al., 2016). Long-term Tai Chi practice has been shown to increase hippocampal volume (Wei et al., 2013). Besides, Tai Chi was associated with improved cerebral blood flow velocity, which may help inhibit the formation and aggregation of Aβ plaques and intracellular NFTs (Burdette et al., 2010).

### Regulate brain inflammatory factors

Inflammation has been implicated in the pathogenesis of chronic neurodegenerative diseases, such as MCI. A meta-analysis has verified several inflammatory markers with significantly different expression levels in patients with MCI compared with healthy controls, such as elevated levels of soluble tumor necrosis factor receptor 2 and interleukin-6 (IL-6; Shen et al., 2019). Some inflammatory markers, such as IL-1 and IL-1β, are directly correlated with the impairment of hippocampal-dependent memory processing (Shaftel et al., 2008). The results of studies focusing on the effects of Tai Chi on inflammation control were inconsistent. Some of them showed reduced levels of IL-6, tumor necrosis factor, and C reactive protein in the Tai Chi group, but some showed no significant changes or even reversed results (Bower and Irwin, 2016). This might be attributed to the complexity of inflammation regulation and the instability of circulating inflammatory markers that can be influenced by many factors apart from Tai Chi. However, RCTs that examined the effects of Tai Chi on inflammation-related gene expression consistently demonstrated reduced activity of nuclear factor kappa B (NF-κB; Bower and Irwin, 2016). NF-κB plays a key role in cognitive performance. A mice experiment indicated that lipopolysaccharides could induce cognitive impairment and neuroinflammation by activating the NF-κB signaling pathway (Zhao et al., 2019). Moreover, another study conducted on Wistar rats found that aerobic exercise could attenuate age-related memory decline, decreased hippocampal NF-κB levels, and atrophy (Lovatel et al., 2013).

**Figure 3:**
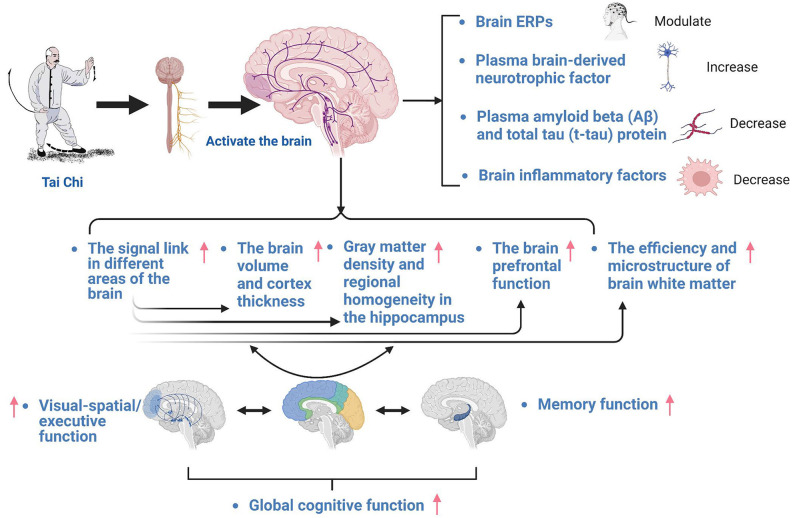
The brain function mechanisms of the Tai Chi treatment for mild cognitive dysfunction.

Although many epidemiological studies have identified the beneficial effects of Tai Chi on various cognitive functions, the underlying neural mechanisms have not been investigated in detail. The performance of cognitive processes depends on the condition of a neural substrate. Previous studies have linked cognitive impairment with structural, neuroelectric, and metabolic abnormalities in a range of brain regions, including the hippocampus, prefrontal cortex, and temporal gyrus. Tai Chi might alleviate cognitive decline through altering factors related to these aspects, particularly by altering the functional connectivity of different brain regions, increasing the activation of the prefrontal cortex and hippocampus, changing brain event-related potential (ERP), and regulating brain inflammatory factors (Figure 3).

### Current problems and prospects

Though guidelines have recommended exercise as an effective nonpharmacologic treatment for preserving cognitive functions in patients with MCI, many older adults are prevented to perform exercises because of physical frailty. Tai Chi may be appealing to elders with MCI because it is a light-to-moderate-intensity aerobic exercise that is safe and friendly to those with different levels of mobility. Furthermore, Tai Chi does not require special equipment or a venue and can thus be performed anywhere at any time.

Tai Chi has its unique advantages over other forms of exercises in improving cognitive functions. First, Tai Chi has many movements. In contrast to general exercises consisting of simple and repetitive movements, Tai Chi requires learning and memorizing different movements and their sequences and formulas, which leads to particularly significant improvements in mental flexibility and immediate recall (Lim et al., 2019). Second, Tai Chi, considered a moving meditation, has a higher level of intellectual involvement. Meditation has been demonstrated to consistently alter some brain regions that are keys to meta-awareness, memory consolidation and reconsolidation, and emotion regulation (Fox et al., 2014). This may shed light on why Tai Chi has stronger effects than general aerobic exercises on brain plasticity and executive functions (Cui et al., 2019; Chen et al., 2020).

Tai Chi has many types and can be classified either by founding family (Chen style, Yang style, Wu Hao style, Wu style, and Sun style) or by the degree of difficulty (8-style, 16-style, 24-style, 32-style, and 42-style). The stance can be performed at three heights (high, medium, and low). The different types or movements of Tai Chi may require different levels of physical strength and flexibility. Selecting a style that works best for one’s condition under the guidance of professionals is important to safety, especially for elders with chronic conditions or disabilities. For example, the lower the stance is, the more power, flexibility, and capacity of balance are needed. Starting Tai Chi from a high stance and gradually moving towards lower ones is preferred for senior beginners. Moreover, the effects of Tai Chi on MCI may vary with style. Most studies used 24-style Tai Chi as the exercise intervention program. Whether other styles can result in better performance is currently unclear. A systematic review indicated that the Yang style tended to be more effective than Sun-style in preventing falls in elders (risk ratio: 0.61 vs. 0.88). More RCTs comparing various Tai Chi styles are needed to determine the most effective one.

Apart from style, duration and frequency may influence the effect size of Tai Chi on MCI. A bibliometric analysis from 2010 to 2020 showed that in most trials with positive effects, Tai Chi was performed for 60 min per time and three times per week for at least 12 weeks (Yang et al., 2021). A recent study speculated that an intervention lasting 6 months to 1 year might lead to further improvements in cognitive functions (Lim et al., 2019). The effect size of Tai Chi on static steady-state balance in older adults was positively correlated with exercise frequency in a meta-analysis (Wang L. C. et al., 2021). Dose-response analyses are needed to optimize the intervention of Tai Chi on MCI.

Tai Chi has been delivered face-to-face by an instructor. Combining Tai Chi with other teaching forms may be an alternative for individuals who prefer to perform Tai Chi at home or who have no access to an instructor. A modified Tai Chi *via* virtual reality has been compared with untrained controls in older adults with cognitive impairment (Hsieh et al., 2018). Significant improvements in cognitive functions were observed in favor of Tai Chi (twice weekly for 6 months) and were closely correlated with the average movement accuracy score. Virtual reality can help practitioners memorize movements by providing verbal and visual cueing and can facilitate standardizing movements by providing immediate feedback.

Though not very consistent, most of the previous epidemiological studies indicated that Tai Chi was a potentially effective strategy for relieving the symptoms of MCI and delaying the progression of MCI to dementia in older adults. The symptoms included but were not limited to memory deficit, executive function decline, sleep disturbance, and body pain. As for the underlying mechanisms, studies demonstrated that Tai Chi can increase connectivity between many brain regions, increase brain volume, and improve the efficiency of the white matter. Additionally, Tai Chi might promote the clearing of neuropathological substrates for MCI and reduce inflammatory factors. Although some of the previous studies appear confusing and even partially contradictory, the combined evidence suggests that Tai Chi may have a positive value in slowing brain aging and rejuvenating brains affected by MCI.

In summary, accumulating evidence from RCTs has suggested Tai Chi as a promising strategy for improving cognitive functions and delaying the onset of dementia in patients with MCI possibly by altering structures and neural activities and regulating other factors (e.g., neurotrophic and inflammatory factors). Future studies are encouraged to compare the different styles of Tai Chi, explore whether a dose-response relationship exists, determine how long an effect can last, and perform subgroup analyses on different types of MCI given that the effects of Tai Chi may vary with specific domain impairment, and investigate potential neural mechanisms by neuroimaging techniques and biochemical markers.

## Author Contributions

XiW and KS contributed to the conception and design of the study. XiW wrote the first draft. XuW and WG organized the research project and corrected the manuscript. All authors contributed to the article and approved the submitted version.
